# Percent change in apparent diffusion coefficient and plasma EBV DNA after induction chemotherapy identifies distinct prognostic response phenotypes in advanced nasopharyngeal carcinoma

**DOI:** 10.1186/s12885-021-09063-1

**Published:** 2021-12-09

**Authors:** Li-Ting Liu, Shan-Shan Guo, Hui Li, Chao Lin, Rui Sun, Qiu-Yan Chen, Yu-Jing Liang, Qing-Nan Tang, Xue-Song Sun, Lin-Quan Tang, Chuan-Miao Xie, Hai-Qiang Mai

**Affiliations:** 1 State Key Laboratory of Oncology in South China; Collaborative Innovation Center for Cancer Medicine; Guangdong Key Laboratory of Nasopharyngeal Carcinoma Diagnosis and Therapy, Sun Yat-sen University Cancer Center, Guangzhou, 510060 China; 2 Department of Nasopharyngeal Carcinoma, Sun Yat-sen University Cancer Center, 651 Dongfeng Road East, Guangzhou, 510060 China; 3 Imaging Diagnosis and Interventional Center, Sun Yat-sen University Cancer Center, Guangzhou, 510060 China

**Keywords:** Nasopharyngeal carcinoma, Apparent diffusion coefficient, EBV DNA, Response phenotypes

## Abstract

**Background:**

To evaluate the prognostic value of the apparent diffusion coefficient (ADC) derived from diffusion-weighted magnetic resonance imaging (MRI) and monitor the early treatment response to induction chemotherapy (IC) with plasma EBV DNA in locoregionally advanced nasopharyngeal carcinoma (LA-NPC).

**Results:**

A total of 307 stage III-IVb NPC patients were prospectively enrolled. All patients underwent MRI examinations to calculate ADC and plasma EBV DNA measurements pretreatment and post-IC. The participants’ ADC value of 92.5% (284/307) increased post-IC. A higher percent change in ADC value (ΔADC%_high_ group) post-IC was associated with a higher 5-year OS rate (90.7% vs 74.9%, *p* < 0.001) than those in the ΔADC%_low_ group. Interestingly, ΔADC% was closely related to the response measured by RECIST 1.1 (*p* < 0.001) and plasma EBV DNA level (*p* = 0.037). The AUC significantly increased when post-IC plasma EBV DNA was added to ΔADC% to predict treatment failure. Thus, based on ΔADC% and plasma EBV DNA, we further divided the participants into three new prognostic response phenotypes (early response, intermediate response, and no response) that correlated with disparate risks of death (*p* = 0.001), disease progression (*p* < 0.001), distant metastasis (*p* < 0.001), and locoregional relapse (*p* < 0.001).

**Conclusion:**

The percentage change in ADC post-IC is indicative of treatment response and clinical outcome. ΔADC% and plasma EBV DNA-based response phenotypes may provide potential utility for early termination of treatment and allow guiding risk-adapted therapeutic strategies for LA-NPC.

**Supplementary Information:**

The online version contains supplementary material available at 10.1186/s12885-021-09063-1.

## Introduction

Nasopharyngeal carcinoma (NPC) is a unique epithelial carcinoma occurring in the nasopharynx. NPC is distinguished from other head and neck cancers with regard to its biological behavior, therapeutic strategies, and etiology coexisting with Epstein-Barr virus infection [1, 2]. Intensity-modulated radiotherapy (IMRT) is the major treatment modality for NPC [3]. For locoregionally advanced NPC (LA-NPC), the curative effects of induction chemotherapy (IC) followed by concurrent chemotherapy (CCRT) have been investigated for the past decade [4–6]. An increasing number of clinical trials have shown that IC is a relatively safe and effective treatment method for LA-NPC that significantly improves clinical outcomes [7, 8]. Benefiting from the development of radiation techniques and multimodality therapies, the survival rate of NPC has favorably improved. However, approximately 20% of individuals still experience distant or locoregional relapse [9, 10]. Thus, early detection of treatment response would enhance existing therapeutic strategies to better optimize outcomes in individuals with a high risk of treatment failure post primary treatment.

Currently, magnetic resonance imaging (MRI) is the dominating imaging tool for diagnosis, staging, and treatment response evaluation in NPC. Diffusion-weighted MRI generates contrast based on the Brownian movement of water molecules restricted by neighboring structures [11, 12]. Quantitative analysis of the diffusion-weighted MRI signal with the apparent diffusion coefficient (ADC) provides a potential imaging marker related to microvascular circulation, membrane integrity, and cell density for tumor characterization and response assessment [13]. The ADC value has been shown to be associated with treatment response and/or outcomes in many malignant diseases, including esophageal cancer, breast cancer, and colorectal cancer [14–16]. Regarding NPC, previous studies demonstrated that ADC increases after IC, and pretreatment ADC or posttreatment changes in ADC are characteristic of treatment response [17–20]. However, the prognostic value and long-term survival prediction of posttreatment changes in ADC in NPC have not been fully investigated.

Therefore, we conducted a prospective study to investigate whether the posttreatment changes in ADC after IC are independent prognostic markers in LA-NPC and explored the clinical significance between the changes in ADC and plasma EBV DNA. This study may be conducive to identifying different responders to IC and potentially guide treatment decisions.

## Methods

All methods were carried out in accordance with SAMPL Guidelines.

### Patients

**Fig. 1:**
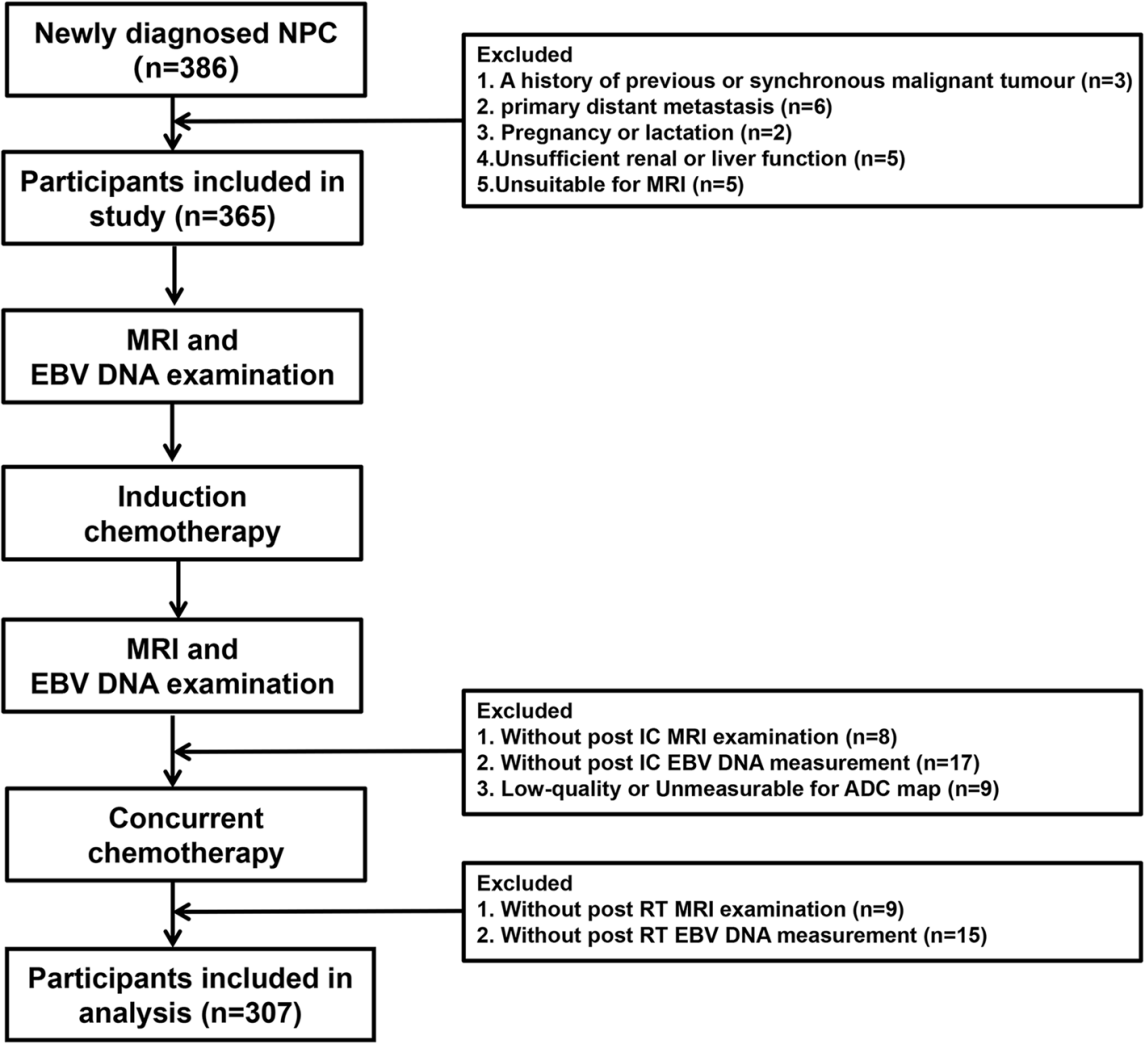
Flowchart of patients included and excluded in this study

This prospective study was conducted in accordance with the principles of the Declaration of Helsinki and approved by the Institutional Review Board (IRB) and Clinical Research Committee of the study institute. Patients were required to provide written informed consent before enrolling in the study. From January 2011 to November 2016, we recruited 386 biopsy-confirmed, newly diagnosed NPC patients. Inclusion criteria are listed as follows: (1) age ≥ 18 years old; (2) stage III-IVb disease according to the seventh edition of the International Union Against Cancer/American Joint Committee on Cancer staging system; (3) score of 0 or 1 on the Eastern Cooperative Oncology Group (ECOG) performance status grade; (4) underwent IC treatment followed by CCRT; (5) complete pretreatment and post-IC plasma EBV DNA measurement data; and (6) adequate function of blood, liver, and kidneys. The exclusion criteria for patients is listed as follows: (1) a history of previous or synchronous malignant tumors, (2) primary distant metastasis, (3) pregnancy or lactation, (4) unsuitability for MRI, (5) no post-IC or RT EBV DNA measurement, and (6) no post-IC or RT MRI examination and low-quality ADC map. A total of 307 eligible participants were included in the final analysis. The case accrual process is summarized in Fig. 1. This study was approved by the Clinical Research Committee of the study institute.

### Study design

All patients were assessed pretreatment through a complete medical history, physical examination, fiber optic nasopharyngoscopy, chest X-rays, abdominal sonography, electrocardiography, and bone scan or 18 F-FDG positron emission tomography/computed tomography scans, hematology, and biochemical profiles. MRI of the nasopharynx, neck examination, and plasma EBV DNA level measurement using real-time quantitative polymerase chain reactions [21] were conducted before treatment, 1 week after completion of IC, and within 1 week after RT. Response was defined radiologically according to the Response Evaluation Criteria in Solid Tumors (RECIST) version 1.1 as complete response (CR), partial response (PR), stable disease (SD), or disease progression (PD) [22]. The response was also evaluated biochemically through plasma EBV DNA levels as detectable (> 0 copies/mL) or undetectable (= 0 copies/mL) cases. The patients who exhibited CR/PR and undetectable EBV DNA post IC were considered to be favorable responders.

### MRI technique

MRIs were conducted using a 3-T imaging technique (Trio Tim; Siemens, Erlangen, Germany). Information on the MRI procedure is detailed in the supplementary materials.

The ADC value was calculated using ADC = −ln [SI(*b*)/SI(0)]/*b*, where SI is the measured signal intensity, b is the b value, and SI(*b*) and SI(0) are the signal intensities with and without diffusion-sensitizing gradients, respectively. Before treatment and post-IC, the ADC value was assessed for both the primary lesions and metastatic lymph nodes on the ADC map at the level of the maximum tumor diameter to cover most of the lesion, avoiding cystic or necrotic components. Regions of interest (ROIs) were defined by selecting areas with high b value DWI (b = 1000 s/mm^2^) and relatively low ADC. Regions with high T2 signal, adjacent adipose and fibroglandular tissue, and biopsy clip artifacts were evaded. Tumor ROIs.

were redefined for each treatment time point, referencing lesion location on pretreatment MRI. The longest diameter of primary tumor was determined on the same largest transverse section in pretreatment MRI and post-IC MRI to assess therapeutic response to IC.

Percentage changes in the ADC values (ΔADC%) were calculated as follows: ΔADC% = (post-IC ADC value - pretreatment ADC value)/pretreatment ADC value × 100%. Each MRI image was analyzed by the same radiologists, and the final regions of interest (ROIs) were checked by another radiologist.

### Treatment

Two to three cycles of induction chemotherapy were administered to all patients, and the regimens of IC included TPF [cisplatin (60–75 mg/m^2^, day 1) and docetaxel (60–75 mg/m^2^, day 1) with 5-fluorouracil (600–750 mg/m2, 96 h of continuous intravenous infusion)] or PF [cisplatin (80–100 mg/m2, day 1) with 5-fluorouracil (800–1000 mg/m2, 96 h of continuous intravenous infusion)]. Concurrent cisplatin (100 mg/m^2^ every 3 weeks) chemotherapy was conducted every 3 weeks at RT. All of the study participants were treated with IMRT, and a simultaneously integrated boost was mandatory. The design of the IMRT plan and technique are detailed in the supplementary materials [23].

### Outcome and follow-up

Overall survival (OS) was the primary study endpoint, which was defined as the time of treatment initiation until death from any cause or last follow-up. Secondary endpoints included progression-free survival (PFS), defined as the time of treatment initiation to the date of the first failure at any site or death from any cause or final follow-up; distant metastasis-free survival (DMFS), defined as the time of treatment initiation to the date of distant relapse or last follow-up; and locoregional relapse-free survival (LRFS), calculated from the time of treatment initiation to locoregional relapse or last follow-up. After treatment, the patients were assessed at least every 3 months for the first 3 years and underwent follow-up examinations every 6 months thereafter or until death.

### Statistical analysis

Details of the statistical analysis procedure are presented in the supplementary materials.

## Results

**Fig. 2:**
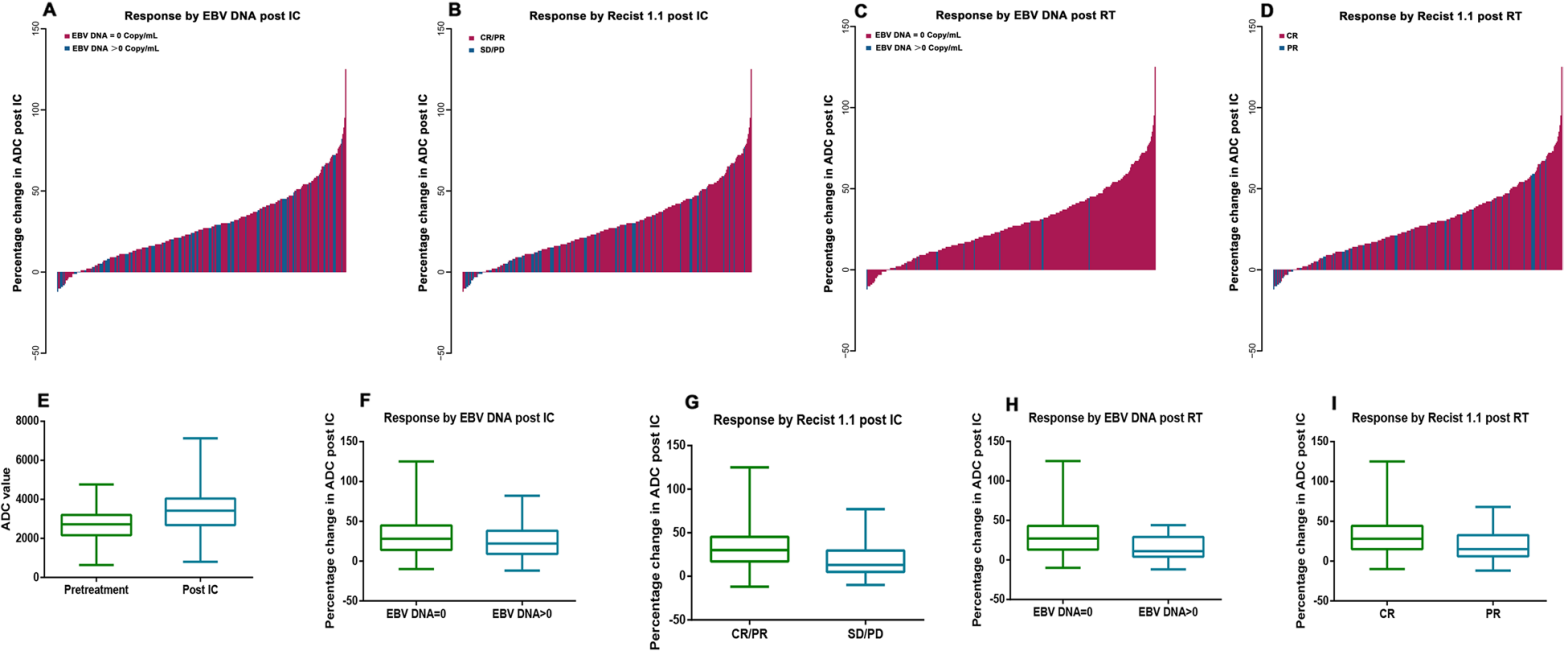
Waterfall plots and box plots showing the association of the percentage change in ADC post induction chemotherapy (IC) with response measured by RECIST 1.1 and plasma EBV DNA levels. (**A**, **D**: percentage change in ADC post IC measured by plasma EBV DNA levels post IC; **B**, **G**: percentage change in ADC post IC measured by response measured by RECIST 1.1 post IC; **C**, **H**: percentage change in ADC post IC measured by plasma EBV DNA levels post radiotherapy; **D**, **I**: percentage change in ADC post IC measured by response measured by RECIST 1.1 post radiotherapy; **E**: The median and range of pretreatment and post IC ADC value)

Of the entire cohort of 307 participants, the median pretreatment ADC value was 2590 × 10^− 6^ mm^2^s^− 1^ (range: 641 × 10^− 6^ mm^2^s^− 1^ - 4420 × 10^− 6^ mm^2^s^− 1^) (Fig. 2E). After IC, the ADC value of 92.5% (284/307) of the participants increased (Fig. 4). The median post-IC ADC value was 3317 × 10^− 6^ mm^2^s^− 1^ (range: 804 × 10^− 6^ mm^2^s^− 1^-5764 × 10^− 6^ mm^2^s^− 1^) (Fig. 2E), and the median percentage change in the ADC value was 26% (range: − 12-125%). After a median follow-up of 72 months (range: 3–108 months), 16.9% (52/307) of the patients died, and 28.9% (89/307) developed disease progression. The 5-year OS, PFS, DMFS, and LRFS rates were 86.6, 73.6, 85.3, and 86.6%, respectively.

### Prognostic value of pretreatment ADC and percentage change in ADC in LA-NPC

**Fig. 3:**
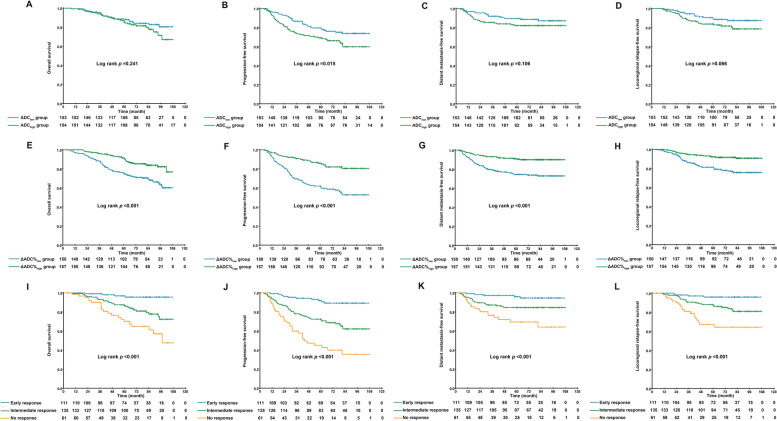
Kaplan-Meier curves of overall survival (OS), progression-free survival (PFS), distant metastasis-free survival (DMFS), and locoregional relapse-free survival (LRFS) of patients with LA-NPC stratified by pretreatment ADC (OS: **A**, PFS: **B**, DMFS: **C**, LRFS: **D**), ΔADC% (OS: **E**, PFS: **F**, DMFS: **G**, LRFS: **H**), and the ΔADC% and plasma EBV DNA-based response phenotypes (OS: **I**, PFS: **J**, DMFS: **K**, LRFS: **L**)

The patients in this study were divided into two groups according to the median pretreatment ADC (ADC_low_ group vs ADC_high_ group) and ΔADC% (ΔADC%_low_ group vs ΔADC%_high_ group). Patient characteristics in the different groups are shown in Table 1. The Kaplan-Meier survival curves of the groups are shown in Fig. 3. For the pretreatment ADC, the 5-year PFS (78.0% vs 68.2%, *p* = 0.015; Fig. 3B) rates for the ADC_low_ group were significantly higher than the corresponding rates for the ADC_high_ group. There were no survival differences in the 5-year OS (87.8% vs 84.5%, *p* = 0.241, Fig. 3A), DMFS (88.6% vs 82.1%, *p* = 0.106, Fig. 3C), or 5-year LRFS (89.5% vs 82.7%, *p* = 0.066, Fig. 3D) rates between the two groups. In terms of ΔADC%, significantly lower survival rates were documented between the ΔADC%_low_ and ΔADC%_high_ groups for all endpoints (OS, 74.9% vs 90.7%, *p* < 0.001, Fig. 3E; PFS, 61.1% vs 85.0%, *p* < 0.001, Fig. 3F; DMFS, 74.4% vs 90.5%, *p* < 0.001, Fig. 3G; LRFS: 80.7% vs 91.6%, *p* < 0.001, Fig. 3H).

**Table 1:** Baseline characteristics

	ΔADC%_low_ Group	ΔADC%_high_ Group	*P* Value	Early Response Group	Intermediate Response Group	No Response Group	*P* Value
Characteristic	No. of patients (%)	No. of patients (%)		No. of patients (%)	No. of patients (%)	No. of patients (%)	
Total	*n* = 150	*n* = 157		*n* = 111	*n* = 135	*n* = 61	
**Age**			0.319				0.683
Median	45	45		45	45	46	
Range	18–77	18–72		22–70	18–74	23–77	
**Sex**			0.744				0.83
Female	33 (22.0%)	37 (23.6%)		24 (21.6%)	33 (24.4%)	13 (21.3%)	
Male	117 (783.0%)	120 (76.4%)		87 (78.4%)	102 (75.6%)	48 (78.7%)	
**T stage**			0.873				0.549
T1	1 (0.7%)	2 (1.3%)		1 (0.9%)	2 (1.5%)	0 (0%)	
T2	14 (9.3%)	18 (11.5%)		13 (11.7%)	15 (11.1%)	4 (6.6%)	
T3	74 (49.3%)	76 (48.4%)		55 (49.5%)	69 (51.1%)	26 (42.6%)	
T4	61 (40.7%)	61 (38.9%)		42 (37.8%)	49 (36.3%)	31 (50.8%)	
**N stage**			0.825				0.314
N0	10 (6.7%)	7 (4.5%)		7 (6.3%)	7 (5.2%)	3 (4.9%)	
N1	54 (36.0%)	59 (37.6%)		43 (38.70%)	53 (39.3%)	17 (27.9%)	
N2	59 (39.3%)	65 (41.4%)		47 (42.3%)	53 (39.3%)	24 (39.3%)	
N3	27 (18.0%)	26 (16.6%)		14 (12.6%)	22 (16.3%)	17 (27.9%)	
**Overall stage**			0.975				0.037
III	72 (48.0%)	75 (47.8%)		58 (52.3%)	70 (51.9%)	19 (31.1%)	
IVa	52 (34.7%)	56 (35.7%)		39 (35.1%)	43 (31.9%)	26 (42.6%)	
IVb	26 (17.3%)	26 (16.6%)		14 (12.6%)	22 (16.3%)	16 (26.2%)	
**Smoking**			0.162				0.078
No	75 (50.0%)	91 (58.0%)		57 (51.4%)	82 (60.7%)	27 (44.3%)	
Yes	75 (50.0%)	66 (42.0%)		54 (48.6%)	53 (39.3%)	34 (55.7%)	
**Family History**			0.978				0.613
No	132 (88.0%)	138 (87.9%)		95 (85.6%)	120 (88.9%)	55 (90.2%)	
Yes	18 (12.0%)	19 (12.1%)		16 (14.4%)	15 (11.1%)	6 (9.8%)	
**Pretreatment ADC_all value (×10**^**−6**^ **mm**^**2**^**/s)**			0.077				0.018
Median	2682.5	2492		2478	2683	2769	
Range	723–4420	641–4076		641–4076	723–4416	1333–4420	
**Post-IC ADC_all value (×10**^**−6**^ **mm**^**2**^**/s)**			< 0.001				0.012
Median	3057.5	3760		3700	3221	3203	
Range	804–4830	965–5764		965–5764	804–5750	1499–4367	
**Pretreatment EBV DNA level**			0.117				< 0.001
< 4000 copies/ml	64 (42.7%)	81 (51.6%)		68 (61.3%)	64 (47.4%)	13 (21.3%)	
≥ 4000 copies/ml	86 (57.3%)	76 (48.4%)		43 (38.7%)	71 (52.6%)	48 (78.7%)	
**Post-IC EBV DNA level**			0.037				< 0.001
= 0 copies/ml	89 (59.3%)	111 (70.7%)		111 (100%)	89 (65.9%)	0 (0%)	
> 0 copies/ml	61 (40.7%)	46 (29.3%)		0 (0%)	46 (34.1%)	61 (100%)	
**Post-RT EBV DNA level**			0.093				
= 0 copies/ml	139 (92.7%)	153 (97.5%)		111 (100%)	129 (95.6%)	52 (85.2%)	< 0.001
> 0 copies/ml	11 (36.7%)	4 (2.5%)		0 (0%)	6 (4.4%)	9 (14.8%)	
**Post-IC response by RECIST 1.1**			< 0.001				< 0.001
CR/PR	91 (63.3%)	131 (83.4%)		101 (91.0%)	95 (70.4%)	26 (42.6%)	
SD/PD	59 (36.7%)	26 (16.6%)		10 (9.0%)	40 (29.6%)	35 (57.4%)	
**Post-RT response by RECIST 1.1**			0.001				< 0.001
CR	109 (72.7%)	137 (87.3%)		101 (91.0%)	108 (80.0%)	37 (60.7%)	
PR	41 (27.3%)	20 (12.7%)		10 (9.0%)	27 (20.0%)	24 (39.3%)	

*Abbreviations*: *ADC* Apparent diffusion coefficient, *ΔADC%* Percentage change in ADC value after induction chemotherapy, *IC* Induction chemotherapy, *RT* Radiotherapy, *CR* Complete response, *PR* Partial response, *SD* Stable diseases, *PD* Disease progression

**Fig. 4:**
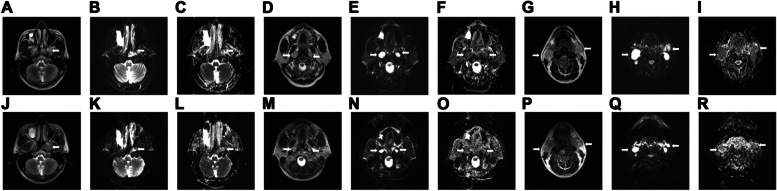
Images of a 47-year-old man with LA-NPC showing intermediate response after induction chemotherapy: (**A**, **D**, and **G**) primary lesion (arrow) in axial T2-weighted MRI with (**B**, **E**, and **H**) corresponding axial diffusion-weighted MRI with b values of 0 and 1000 s/mm^2^ (**C**, **F**, and **I**), apparent diffusion coefficient (ADC) maps pretreatment, and (**J**-**R**) matched sections of the same imaging sequences after induction chemotherapy

A multivariate analysis conducted to adjust for confounding factors demonstrated that ΔADC% was an independent prognostic factor for OS (HR = 0.44, 95% CI = 0.24–0.82, *p* = 0.009), PFS (HR = 0.35, 95% CI = 0.22–0.56, *p* < 0.001), DMFS (HR = 0.42, 95% CI = 0.21–0.81, *p* = 0.010), and LRFS (HR = 0.26, 95% CI = 0.12–0.55, *p* < 0.001) (Table 2). Receiver operating characteristic (ROC) curves used to assess the prognostic validity demonstrated that the area under the curve (AUC) of ΔADC% was significantly higher than that of pretreatment ADC (0.634 vs 0.534, *p* = 0.002, Supplementary Fig. 1). Thus, ΔADC% was a better prognostic indicator for the clinical outcomes than pretreatment ADC.

**Table 2:** Cox proportional hazard analyses of 307 locoregionally advanced nasopharyngeal carcinoma patients

Factors	HR_OS_ (95%CI)	*P* Value	HR_PFS_ (95%CI)	*P* Value	HR_DMFS_ (95%CI)	*P* Value	HR_LRFS_ (95%CI)	*P* Value
**Pretreatment EBV DNA level**^a^	3.21 (1.58–6.53)	0.001	3.11 (1.85–5.23)	< 0.001	2.55 (1.21–5.35)	0.013	3.14 (1.51–6.54)	0.002
**Post-IC EBV DNA level**^b^	2.50 (1.37–4.54)	0.003	2.41 (1.52–3.80)	< 0.001	2.42 (1.26–4.65)	0.008	2.25 (1.19–4.26)	0.013
**Pretreatment ADC value**^a^	0.89 (0.47–1.70)	0.727	1.42 (0.86–2.33)	0.170	1.08 (0.54–2.15)	0.838	1.83 (0.89–3.79)	0.103
**ΔADC%**^b^	0.44 (0.24–0.82)	0.001	0.35 (0.22–0.56)	< 0.001	0.42 (0.21–0.81)	0.01	0.26 (0.12–0.55)	< 0.001
**Response phenotypes**^c^								
Early response	reference	0.001	reference	< 0.001	reference	0.001	reference	< 0.001
Intermediate response	5.50 (1.91–15.82)	0.002	4.80 (2.33–9.88)	< 0.001	3.96 (1.47–10.66)	0.006	4.93 (1.69–14.41)	0.004
No response	8.52 (2.90–25.09)	< 0.001	8.83 (4.19–18.59)	< 0.001	7.03 (2.58–19.19)	< 0.001	10.50 (3.51–31.45)	< 0.001

*Abbreviations*: *HR* Hazard ratio, *CI* Confidence interval, *OS* Overall survival, *PFS* Progression-free survival, *DMFS* Distant metastasis-free survival, *LRFS* Locoregional relapse-free survival, *ADC* Apparent diffusion coefficient, *IC* Induction chemotherapy, *RT* Radiotherapy, *ΔADC%* Percentage change in ADC value after induction chemotherapy; Response phenotypes = response phenotypes based on percentage change in ADC and plasma EBV DNA post IC

^a^Age, sex, T stage, N stage, smoking, family history, pretreatment EBV DNA level and pretreatment ADC value were included in the Cox regression model

^b^Age, sex, T stage, N stage, smoking, family history, post-IC EBV DNA level and percentage change in ADC post IC were included in the Cox regression model

^c^Age, sex, T stage, N stage, smoking, family history and response status were included in the Cox regression model

### Association of ΔADC% with response measured by RECIST 1.1 and plasma EBV DNA levels

The participants in the ΔADC%_high_ group were significantly more likely to achieve CR/PR after IC than those in the ΔADC%_low_ group (83.4% vs 63.3%, *p* < 0.001, Table 1, Fig. 2B and G). Moreover, a significantly higher proportion of the participants in the ΔADC%_low_ group had residual disease after RT than those in the ΔADC%_high_ group (72.7% vs 87.3%, *p* = 0.001, Fig. 2D and I).

The plasma EBV DNA levels of the participants in the ΔADC%_high_ group were more likely to decrease to undetectable levels both post-IC (70.7% vs 59.3%, *p* = 0.037, Fig. 2A and F) and post-RT (92.7% vs 97.5%, *p* = 0.093, Fig. 2C and H) compared with the ΔADC%_low_ group. The ROC curves also showed that the AUC significantly increased when ΔADC% was added to post-IC plasma EBV DNA for predicting treatment failure (Supplementary Fig. 1).

Based on these observations, we defined the patients with three different ΔADC% and plasma EBV DNA-based response phenotypes: early response (ΔADC%_high_ with undetectable EBV DNA post-IC), intermediate response (ΔADC%_high_ with detectable EBV DNA or ΔADC%_low_ with undetectable EBV DNA post-IC), and no response (ΔADC%_low_ with detectable EBV DNA post-IC).

### ΔADC% and EBV DNA-based response phenotypes predicted the prognosis of LA-NPC

Additionally, we evaluated the prognostic association of the three response phenotypes with survival outcomes in our study. We observed that prognoses were significantly different among the groups. In particular, the patients in the early response group achieved the most favorable survival among all endpoints (5-year OS = 95.6, 86.7, and 70.1%, respectively, *p* < 0.001, Fig. 3I; PFS = 90.6, 71.5, and 42.6%, respectively, *p* < 0.001, Fig. 3J; DMFS = 96.0, 84.6, and 69.4%, respectively, *p* < 0.001, Fig. 3K; and LRFS = 95.9, 86.2, and 64.3%, respectively, *p* < 0.001, Fig. 3L).

We investigated potential associations between the ΔADC% and plasma EBV DNA-based response phenotypes and other clinical covariates that may have influenced the outcome, including age, sex, T stage, N stage, smoking, and family history. The response phenotype was an independent prognostic indicator for OS (*p* = 0.001), PFS (*p* < 0.001), DMFS (*p* = 0.001), and LRFS (*p* < 0.001) (Table 2).

## Discussion

In this study, we demonstrated that the percentage change in ADC was a prognostic indicator independent of other clinical factors that could predict and identify responses to induction chemotherapy. The ADC increase in the patients with LA-NPC after induction chemotherapy appeared to be in line with the effective treatment. The patients in the ΔADC%_high_ group were more likely to achieve CR/PR and undetectable EBV DNA after IC than those in the ΔADC%_low_ group. Combining the ΔADC% with plasma EBV DNA after IC resulted in superior prognostic values for clinical outcomes compared to ΔADC% alone.

The establishment of therapeutic decisions is primarily based on the TNM stage. However, the TNM stage reflects only the anatomical invasion of tumors and lacks biological information, as clinical outcomes vary substantially among patients with the same stage. Diffusion-weighted MRI signals using ADC enable noninvasive assessment of tumor microstructure and the intrinsic biological characteristics of tissues and have proven value for monitoring response to therapy and predicting prognosis in many malignancies [13–15]. Low ADC values have been shown to indicate viable tissue with high cellularity based on the random displacement of water molecules, with increased ADC posttreatment reflecting increased extracellular space [24, 25].

In 2003, a study demonstrated that changes in diffusion parameters 1 week after initiating treatment using DW-MRI could detect early tumor response to RT in brain malignancies [26]. Later, breast cancer studies also reported that the percent change in ADC for assessing early tumor response to IC than morphological variables [27] and the change in ADC predicted complete pathologic response to IC [15]. A recent multicenter study in ovarian cancer showed that ADC changes are indicative of tumor response. After IC, increased ADC is indicative of improved progression-free survival in relapsed disease, suggesting its potential for effective treatment management.

Previous studies of NPC found that pretreatment ADC was a valuable marker for the prediction of the response to IC and local failure [17, 19]. Regarding the change in ADC, Chen et al. showed that a large increase in ADC was associated with good treatment response after IC in a cohort study of 31 patients with LA-NPC [18]. Zhang et al. and Hong et al. also found that percentage increases in ADC after IC were higher for responders than for nonresponders [20, 28]. In our study, post-IC percentage changes in ADC correlated with response evaluated by RECIST 1.1, which was in line with the results of previous studies. However, the relationship between the percentage change in ADC and clinical outcomes was not explored. The correlation between post-IC percentage changes in ADC and long-term survival outcomes in NPC remains unknown. Hence, we further analyzed the association of post-IC percentage change in ADC with clinical outcomes in a large cohort of patients with NPC from the endemic region and demonstrated that post-IC percentage change in ADC was an independent prognostic factor for overall survival, progression-free survival, distant metastasis-free survival, and locoregional relapse-free survival. The post-IC percentage change in ADC was observed to be closely related to the plasma EBV DNA change, a biological marker that has been widely used to predict the treatment response and prognosis of NPC [29, 30]. Consistent with these observations, we further divided the patients in this study into three different response phenotypes: early response, intermediate response, and no response. Notably, our results confirmed that the aforementioned phenotypic groups were associated with disparate risks of death, disease progression, and locoregional and distant relapse. Thus, prognostic response phenotypes could provide directions for future clinical trial designs. For patients in the early response groups, the treatment intensity could be reduced to avoid unnecessary toxicities. Therefore, we suggested reducing the radiation dose of RT, by either substituting concurrent chemotherapy with EGFR inhibitors or sparing concurrent chemotherapy with RT. Subsequently, for patients in intermediate groups, we proposed exploring the integration of EGFR inhibitors and immune checkpoint inhibitor therapy with CCRT. Finally, the combination of a second antitumor drug, such as paclitaxel, to enhance the radiosensitivity of CCRT, the inclusion of adjuvant chemotherapy, or the addition of immunotherapy for patients in the no response group can target residual resistant disease.

This study had limitations. First, the participants were collected from only a single center, and the results may not be easily generalized to other centers as a result of interinstitutional differences in MRI techniques. Second, no standard method was established to determine the ADC value. Thus, the applicability of the results in more challenging cohorts remains to be investigated. Prospective multicenter studies are required to validate the results of our study.

## Conclusion

ADC measurements provide a noninvasive method of detecting early microstructural changes that occur in response to LA-NPC treatment. When there was residual measurable disease and plasma EBV DNA, ADC changes were greater in responders than in nonresponders. We identified patients with different radiobiological responses with disparate relapse risks among LA-NPC treated with IC followed by CCRT. These response phenotypes may allow different treatment intensities for optimal tumor control. As the initial treatment is important for LA-NPC, determining the appropriate treatment is crucial. The capacity to predict response may enable early intervention of treatment in nonresponding patients, avoid supererogatory toxicity, and allow early changes in therapeutic strategy.

## Supplementary Information

**Additional file 1.**

## Data Availability

The datasets generated and/or analyzed during the current study are not publicly available due to the privacy of the patients but are available from the corresponding author on reasonable request.

## Abbreviations

ADC: Apparent diffusion coefficient
ΔADC%: Percentage change in ADC value
CCRT: Concurrent chemoradiotherapy
CR: Complete response
DMFS: Distant metastasis-free survival
EBV DNA: Epstein-Barr virus deoxyribonucleic acid
IC: Induction chemotherapy
IMRT: Intensity modulated radiation therapy
LA-NPC: Locoregionally advanced nasopharyngeal carcinoma
LRFS: Locoregional relapse-free survival
MRI: Magnetic resonance imaging
NPC: Nasopharyngeal carcinoma
OS: Overall survival
PFS: Progression-free survival
PD: Disease progression
PR: Partial response
RECIST: Response Evaluation Criteria in Solid Tumors
ROC: Receiver-operating characteristic
ROIs: Regions of interest
SD: Stable disease
SI: Signal intensity
