# Supplementary material for: Percent change in apparent diffusion coefficient and plasma EBV DNA after induction chemotherapy identifies distinct prognostic response phenotypes in advanced nasopharyngeal carcinoma
Source: BMC Cancer. 2021 Dec 9;21:1320. doi: 10.1186/s12885-021-09063-1 (PMC8662833; doi:10.1186/s12885-021-09063-1)

**Statistical analysis**

Categorical variables were compared using the χ^2^ test or Fisher’s exact test. Actuarial rates were calculated using the Kaplan-Meier method and survival curves were compared using the log-rank test. Multivariate analyses using a Cox proportional hazards model were used to test the independent significance of potential prognostic factors. SPSS version 24.0 (SPSS, Chicago, IL, USA) and R version 3.5.0 ([www.r-project.org](http://www.r-project.org)) were used for statistical analyses. A two-tailed *p* value < 0.05 was considered statistically significant.

**MRI technique**

MRIs were conducted using a 3-T imaging system (Trio Tim; Siemens, Erlangen, Germany). The following MRI sequences were used: T1-weighted fast spin-echo images without contrast in the axial, coronal, and sagittal planes and T2-weighted fast spin-echo images in the axial plane in a spin-echo echo-planar DWI sequence (matrix, 192 × 192; TR/TE, 5100 ms/96 ms; fov, 240; b values, 0 and 1000 s/mm2; three signal averages) obtained before contrast injection. After intravenous gadopentetate dimeglumine injection at a dose of 0.1 mmol/kg of body weight, spin-echo T1-weighted axial and sagittal sequences and spin-echo T1-weighted fat-suppressed coronal sequences were obtained. All of the patients were scanned from the vertex through the clavicles with a 5 mm thick and 1 mm interslice gap for the axial plane and a 6 mm thick and 1 mm interslice gap for the coronal and sagittal planes.

**Supplementary table 1. AUC value for the prediction of prognosis for pre-treatment ADC, ΔADC%, and ΔADC% and plasma EBV DNA based response phenotypes of 307 locoregionally advanced nasopharyngeal carcinoma patients.**

|  | **Death** | |  | **Disease progression** | |  | **Distant metastasis** | |  | **Locoregional relapse** | |
| --- | --- | --- | --- | --- | --- | --- | --- | --- | --- | --- | --- |
| **Factors** | AUC | *p* value |  | AUC | *p* value |  | AUC | *p* value |  | AUC | *p* value |
| **Pretreatment ADC** | 0.534 | 0.443 |  | 0.566 | 0.069 |  | 0.560 | 0.208 |  | 0.573 | 0.123 |
| **ΔADC%** | 0.634 | 0.002 |  | 0.662 | <0.001 |  | 0.622 | 0.011 |  | 0.676 | <0.001 |
| **Response phenotypes** | 0.716 | <0.001 |  | 0.723 | <0.001 |  | 0.696 | <0.001 |  | 0.707 | <0.001 |

Abbreviations: **ΔADC%** = Percentage changes in apparent diffusion coefficient; **IC** =induction chemotherapy; **RT** =radiotherapy; **Response phenotypes** = response phenotypes based on percentage change in ADC and plasma EBV DNA post IC

**Supplementary figure 1:** Receiver-operating characteristic curves for the prediction of death (A), disease progression (B), distant metastasis (C), and locoregional relapse (D) for pre-treatment ADC, ΔADC%, and the combination of ΔADC% and plasma EBV DNA post-IC in patients with LA-NPC.


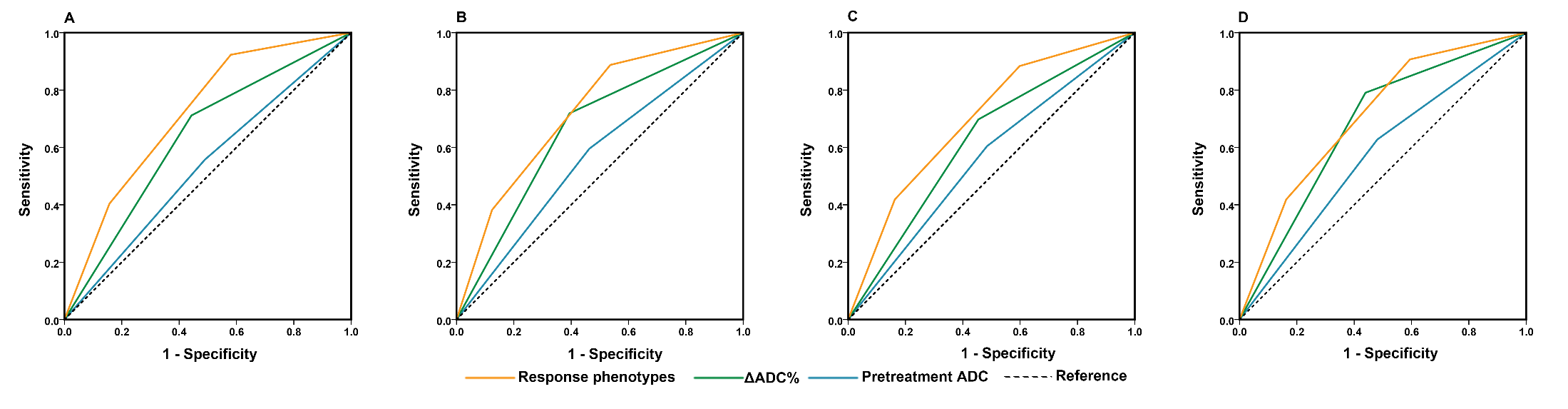

Supplement: Supplementary file 1 — Additional file 1. [file 12885_2021_9063_MOESM1_ESM.docx]
